# Exploring the implementation of tobacco cessation services in government healthcare facilities, Maharashtra: a qualitative approach

**DOI:** 10.1186/s12913-026-14499-x

**Published:** 2026-04-17

**Authors:** Nilesh Gawde, Zahiruddin Quazi Syed, Subita Patil, Umesh Shirodkar

**Affiliations:** 1 Centre for Public Health, School of Health Systems Studies, Tata Institute of Social Sciences, Mumbai, 400088 India; 2 Department of Community Medicine, Jawaharlal Nehru Medical College, Datta Meghe Institute of Higher Education and Research, Wardha, 442005 India; 3 Department of Preventive Oncology, Tata Memorial Hospital, Mumbai, 400012 India; 4 Commissionerate of Health, Government of Maharashtra, Mumbai, 400001 India

**Keywords:** Tobacco use cessation, Health services research, Process assessment, India, 5A model

## Abstract

**Background:**

India is the second-largest consumer of tobacco. Tobacco cessation clinics were introduced in India in 2001 and have been decentralised to the district level through the National Tobacco Control Programme. Research evidence on effective cessation interventions is limited in India.

**Objectives:**

This paper aims to explore the implementation of tobacco cessation services in the public sector at the district level from a provider perspective.

**Methods:**

Two Maharashtra districts with a high prevalence of tobacco use were selected. Healthcare providers responsible for tobacco cessation services at the tertiary, secondary and primary levels were purposively selected. An exploratory qualitative study design was employed. Linnan and Steckler’s process evaluation framework guided the design and analysis of the data.

**Results:**

Resources are inadequate to meet the requirements within districts, resulting in a weak ‘reach’ of services to tobacco users. The utilisation of follow-up services is limited except for those with chronic conditions. Among the 5 A’s, ‘Ask’ and ‘Advise’ are common, but the remaining three are used only by select healthcare providers. Data on cessation outcomes are limited, if at all. Healthcare providers reported that cessation outcomes were poor.

**Conclusions:**

The reach of tobacco cessation services is limited, and there is a need to increase the capacity of primary-level providers with brief key messages. The ‘fear-inducing’ tone is predominant in the ‘Advice’ messages. This needs to be supplemented by ‘Assist’ and ‘Arrange’ approaches. Cessation outcomes should be systematically recorded in health service systems.

**Supplementary information:**

The online version contains supplementary material available at https://doi.org/10.1186/s12913-026-14499-x.

## Introduction

The tobacco use pandemic has resulted in millions of deaths, with an estimated 8.7 million deaths annually in 2019 alone [1]. Nearly one-fifth of all-cause mortality among men is attributable to smoking alone [2]. With more than one billion users globally, millions of deaths are expected to occur in the coming years. Most of these deaths are expected in low- and middle-income countries (LMICs), where 80% of tobacco users live. India alone had 266 million adult tobacco users (nearly a quarter of the global users) in 2016–17 [3].

The Global Noncommunicable Disease Action Plan has set a 40% relative reduction in tobacco use as its 2030 target [4]. Offering help to tobacco users to quit, the “O” in WHO’s MPOWER strategy is a crucial intervention. However, implementation of the WHO Framework Convention on Tobacco Control (FCTC) and its MPOWER, including the “O” measure, varies across countries. It is well established that quitting tobacco results in health benefits, preventing premature death and improving the quality of life of individuals [5]. At the societal level, cessation interventions are cost-effective and reduce healthcare expenditures. WHO defines comprehensive tobacco cessation as a national quitting line and coverage of both nicotine replacement therapy (NRT) and counselling services, with costs covered. In 2022, approximately one-third of the global population in 32 countries had access to comprehensive tobacco cessation services, and half of the persons covered were from India alone [6]. Although India is a better performer than its neighbours, tobacco cessation services are available only in some facilities, and the implementation challenges have not been thoroughly examined.

Most of the literature on the effectiveness of tobacco cessation interventions is from high-income countries. Most of the RCTs in India had counselling or health education as interventions, and very few were drug trials [7]. There has yet to be an RCT on Quitline or mobile-based cessation interventions in India, although observational studies have documented success [8, 9]. A systematic review focusing solely on behavioural interventions in India reported good efficacy for tobacco users [10]. The interventions tested were heterogeneous, comprising individual, group, or both interventions. There was also variation concerning content, duration, and intervention method, which were attempted in diverse populations. While RCTs provide some evidence of efficacy in India, best practices are yet to be established.

The new millennium witnessed the dawn of tobacco cessation clinics (TCCs) in India, which were predominantly in tertiary care facilities [11]. TCCs offered a relatively standard package of services, unlike the diversity of interventions in the research studies. The results in practice were often far short of the promise seen in trial settings, and therefore, it gave low quit rates by these TCC [12]. Recent evidence suggests an improved quit rate with follow-up through a quit line approach [13]. TCCs were decentralised to the district level (Secondary level) under the National Tobacco Control Programme, but a recent review noted that the district TCCs were ill-equipped, providers were not actively referring cases to cessation facilities, interventions had weak follow-up mechanisms, and poor data quality [14]. Yet, the challenges of reaching tobacco users, adherence to standard therapeutic approaches in practice, client compliance, and the strength of follow-up mechanisms have not been documented in practice settings.

Non-communicable disease clinics have shown promise for introducing tobacco cessation services, and one trial has demonstrated their effectiveness [15, 16]. The NCD staff have initiated delivery of tobacco cessation services at the secondary and primary levels. However, there is no literature that documents the implementation challenges in such a decentralised model of healthcare.

Experimental research in Indian settings has identified successful models in specific contexts, but the best practices for tobacco cessation services in diverse epidemics are still in the early stages of development. Tobacco cessation services in Indian healthcare settings face resource and capacity constraints, and the follow-up rates among clients are not encouraging [17]. There is a need for implementation science research techniques, such as process evaluations, to assess the implementation of cessation services and identify factors that influence it. The implementation of cessation interventions at the secondary and primary level (district level and below) remains unexplored.

The second round of India’s Global Adult Tobacco Survey (GATS) reported use of pharmacotherapy by 4.1% smokers and 3.2% smokeless tobacco users who attempted quitting and counselling services by 8.6% smokers and 7.3% smokeless tobacco users [3]. This quantitative data is captured from the clients’ perspective. The providers’ perspective, which could detail the implementation bottlenecks, has not been studied, especially at the secondary and primary care levels. A recent review highlights the weak delivery of cessation services at the secondary care level and within the national health programmes and calls for strengthening of cessation services throughout the healthcare delivery systems [18].

Maharashtra is the second-highest populous state of India, with tobacco use prevalence similar to the national level [3]. The state has relatively stronger healthcare delivery systems and has been implementing the National Tobacco Control Programme for more than a decade. Currently, all 36 districts provide tobacco cessation services. Hence, the state provided an appropriate context to study the implementation of cessation services. Maharashtra’s public healthcare system comprises three tiers: Primary Health Centres (PHCs) and Health and Wellness Centres (HWCs) at the primary level; rural, sub-district, and district hospitals at the secondary level; and medical colleges and other specialised hospitals at the tertiary care level. The structure of healthcare delivery systems is the same in tribal and non-tribal districts. However, the tribal districts have a higher number of healthcare facilities per capita. For example, one PHC is expected to cater to a population of 30,000 in rural areas, but only 20,000 in tribal areas.

Health services in the district continue the legacy of vertical programmes. Counselling services are provided at health facilities for HIV, TB, tobacco, Non-Communicable Diseases (NCDs), mental health, the *Rashtriya Kishor Swasthya Karyakram* (RKSK, an adolescent health programme), and the District Mental Health Programme. India’s community volunteers, Accredited Social Health Activists (ASHA), are entrusted with responsibilities for health education and community action. They work under close supervision and support from auxiliary nurse midwives (ANMs) and community health officers (CHOs) of the HWCs, who have preventive services, including health education, as their primary responsibility.

Secondary data from GATS 2016-17 from Maharashtra revealed that less than half of the smokers (45.7%) and less than one-third of the smokeless tobacco users (30.2%) were asked to quit by healthcare providers during the previous 12 months. Only approximately one-fifth of smokers and smokeless tobacco users reported quit attempts in the preceding 12 months. Among those who attempted to quit, very few smokers and smokeless tobacco users used pharmacotherapy (9.5% and 5.4%, respectively) or counselling (3.1% and 5.4%). The survey, however, does not provide any information on the implementation structures and bottlenecks to the tobacco cessation services.

This paper aims to study the implementation of the tobacco cessation services from the providers’ perspective at the secondary and primary care levels within the health systems of Maharashtra. The paper examines the resources and processes employed within health systems and the impressions of healthcare providers on the resultant outcomes, capturing the narratives of healthcare providers.

## Methods

Study design: This paper is part of a larger study that assessed the implementation of NTCP in two tribal districts of Maharashtra. The provider perspective component of the study used an exploratory qualitative design. The NTCP implementation framework provided the basis for the programme theory, and we used Linnan’s and Steckler’s process evaluation framework to examine the implementation process. The qualitative exploration focussed on resources and processes employed as narrated by the providers, especially the ‘how’ of the cessation services and the resultant outcomes as per the providers’ impressions.

Sampling of districts: The latest district-wise tobacco use indicators were taken from the National Family and Health Survey–V (NFHS-V, 2019-21). The top four districts featured on both lists were *Gadchiroli*,* Bhandara*,* Gondiya*, and *Nandurbar*. Since the first three districts belong to the same geographical region of Vidarbha, with similar cultures and health systems, one of them, namely *Gadchiroli*, was purposively selected. *Nandurbar*, located in North Maharashtra, was the second district included in the study.

Selection of participants: Healthcare service providers at all three levels of care (primary, secondary and tertiary) formed the universe. Healthcare providers who were assigned responsibility for tobacco cessation services were purposively selected. In both districts, the staff of the tobacco cessation centre (Tertiary), counsellors in other national health programmes at the secondary and tertiary levels, and health workers at the primary level who provided cessation advice to patients accessing health services were selected. The sample included all staff members of the tobacco cessation centres and all counsellors working in the NCD and RKSK programmes. The other workers were purposively selected. A total of 57 providers (40 from four focus group discussions and 17 from in-depth interviews) participated in the study, with one provider declining to participate. At that time, similar responses were getting repeated, and it was decided to stop recruitment on the basis of data saturation.

The first author is a faculty member at one of universities in India and holds an MD in Preventive and Social Medicine. He conducted all the interviews and the focus group discussion. He had undergone formal training in qualitative research methodologies, including workshops and formal coursework, and has led qualitative research projects. The researcher visited district health authorities (Civil surgeon and District Tobacco Control Programme officer) in both districts a month prior to actual data collection and explained the purpose of the research. Potential participants were informed in advance about the research, and their prior appointment was taken. The researcher was a member of the second round of India’s Global Adult Tobacco Survey, which led to this area of interest for doctoral study, and this was shared with the participants.

Data collection and analysis: An in-depth interview guide was designed to capture the provider’s perspective. This interview guide was also used for the focus group discussions with block-level NCD and RKSK counsellors who were also providers. A second interview guide was developed for other stakeholders. Both interview guides are available as supplementary material 1. The 5As were added as probes after pilot testing. The interviews were conducted face-to-face, and audio recorded; the recordings were later transcribed. Field-level data collection lasted from March 2023 to May 2023. All interviews were conducted in separate rooms at the healthcare facilities, ensuring privacy and confidentiality, typically in the afternoon when workers would be free from direct client services. The interviews lasted between 23 min and 1 h 26 min.

A draft codebook was prepared based on the conceptual framework, which was revised following the reading of the first few transcripts. The Atlas.ti 24 version was used for coding by the first author. Key themes and messages were identified by reviewing the transcripts and the field notes by the first author. Linnan’s and Steckler’s process evaluation frameworks were used to capture the implementation of tobacco cessation interventions (context, resources, reach, fidelity, dose, and outcomes) from the providers’ perspective [19]. Context was defined the larger social, political, and economic environment that may influence the cessation services. Resources include all resources, administrative structures, policies, regulations, and incentives for the cessation services. Reach referred to the proportion of the intended target audience that received cessation services. Fidelity focused on the 5As of cessation counselling. Intensity (Dose) referred to the number of intended units of cessation services (frequency and duration) received by the client. Outcomes referred to the cessation attempts and their sustenance. The statements that conveyed the 5 As approach (Ask, Advise, Assess, Assist, and Arrange) were identified from the transcripts. The Maharashtra GATS data (2016–17) were processed to obtain state-specific cessation indicators.

The Institutional Ethics Committee of the Datta Meghe Institute of Medical Sciences approved the study protocol in August 2019. The study followed ethical principles of autonomy, privacy and confidentiality. There was no direct benefit to participants in this observational study. However, the data collection process brought together ideas, especially through group discussions.

## Results

The study participants included six staff from TCCs, two from the Tata Memorial Hospital project, three from TB, one from HIV, two from NCD, two from RKSK, and one from a tertiary level. The secondary level included NCD and RKSK teams (20 members each). The primary-level participants included two medical officers, four ANMs, and seven ASHAs.

Context: Tobacco use was widespread in both tribal districts. There are numerous tobacco-selling points across villages. The local vendors sell packaged and freshly prepared tobacco. Freshly prepared *Kharra* (tobacco, lime, areca nut, and paan masala mixture) was consumed most commonly. *Gudakhu*, a dental product, is common among women.

*“Both alcohol and tobacco are part and parcel of their (Tribal communities in three blocks along Narmada) life. All festive occasions*,* such as marriages*,* childbirths*,* and funerals*,* ceremonially use tobacco and alcohol. This provides an avenue to fuel the epidemic: less control and more spread. The husband and wife both consume tobacco*,* and if their infant is as young as 6 months if cries*,* they are given tobacco”*, an NCD counsellor. The families offered *Kharra* to guests, and young children were sent to local vendors to fetch it. In some villages, parents give tobacco products to school-going children; it was seen almost as a food ration item.

The health workers reported rampant use of promotional techniques by the tobacco industry, including surrogate advertising of paan masala brands by celebrities through television, hoarding, and free clothing bags and other products with the brand logos of tobacco companies.

### Resources and training

Both districts had tobacco cessation clinics (TCCs) at the respective district hospitals (secondary level), with one counsellor and a social worker under the leadership of a district consultant. The team had NTCP execution responsibilities, including administrative and outdoor activities. Therefore, TCC services operated intermittently at *Nandurbar*. Tata Memorial Hospital, a premier cancer treatment and research institute in India, provided additional human resources to *Gadchiroli* TCC, ensuring daily operations.

The responsibility for tobacco use screening and cessation counselling was entrusted to counsellors under the noncommunicable disease (NCD) control programme at rural hospitals (secondary care level). There were 13 hospitals at the secondary level in *Gadchiroli* and 14 in *Nandurbar* (including district, sub-district and rural hospitals). Both districts had about 50 PHCs (45 in *Gadchiroli* and 58 in *Nandurbar*), where the responsibility rested with the PHC medical officer. The state organises annual training for TCC staff covering all NTCP responsibilities. The emphasis on cessation counselling was limited. Among the six TCC staff members interviewed, only one could state the approach of A5. None could describe the 5 Rs, the transtheoretical model, motivational interviewing or cognitive behaviour therapy. Table 1 details the resources in health systems, including training in tobacco cessation.

**Table 1:** Resources and training

Resources	Primary care level	Secondary care level
Facility names	Primary Health Centre (PHC), Health and Wellness Centre (HWC)	Rural Hospital (Community Health Centre – CHC) and District Hospital (DH)
NCD programme	Not applicable	One counsellor, training at the district level
Tuberculosis (TB) programme	Medical officers, training in TB	Medical officer and Senior TB treatment supervisor, counselor training in TB
RCH staff	ASHA, ANM, medical officer, orientation at block level	Medical officer, staff nurse, orientation at block and district level
RKSK programme	Not applicable	One counsellor, orientation at the district level
AIDS programme	Not applicable	Medical Officer, Counselor, training in HIV
Tobacco Cessation Centre	Not applicable	Three-member team, trained at State
Other programmes and general health staff	Resources not trained in tobacco cessation	Resources not trained in tobacco cessation

Pharmacotherapy: *“Nicotine chewing gums were available in 2018 within NTCP. It was given only for one month to a patient because the purpose was to help in the transition from tobacco to no tobacco to no nicotine.”*-District official. Only nicotine replacement therapy was available in the past.

### Reach

Tobacco cessation services were offered to patients and clients visiting health systems. At the tertiary and secondary care levels, there was a systematic screening of all adults aged above 30 years for noncommunicable diseases, including ‘Asking’ the patients about tobacco use. Table 2 details the reach of tobacco cessation’s first A (Ask).

**Table 2:** Reach with respect to ‘Ask’ tobacco cessation service

Type of client	Primary care level	Secondary care level
All persons aged 30 and above visiting the facility	Few	Nearly all
NCD patients	Few	Nearly all
Tuberculosis	All	All
Pregnant women	Most	Nearly all
Other patients	Few	Few

*“We have made a stamp. That stamp is put up on case paper at counter number one. The stamp has a point on tobacco use with a remark of yes or no. If any patient gets* ‘*yes*’ *on the paper*,* that patient is sent to TCC”*-TCC staff.

However, not every person who was ‘Asked’ reached the TCC for an ‘Advice’. The *Nandurbar* TCC records 100–150 patients monthly, whereas the *Gadchiroli* TCC’s reach is approximately 400 patients monthly.

*“If 15 patients are referred by the NCD OPD*,* 4 to 5 patients reach the TCC. If the patients are told that tobacco counselling is to be done*,* then they take a U-turn from there and do not reach it here. When a patient comes to a dental OPD with a white patch*,* red patch*,* or swelling and recognises its seriousness*,* they come to the TCC. They try to obtain complete information. Most people coming to the TCC are aged 40. There are very few individuals aged 20–40 years. Young people do not face symptoms (hence reluctance); when they exceed 40 years of age*,* they develop NCDs or oral lesions*,* and then they tend to develop symptoms. We try to reach out to all. One who is having symptoms or difficulty due to tobacco use comes*,* and those who do not have symptoms or difficulty do not turn up. (They ask) What is this? Our parents and ancestors used it throughout their lives; they did not have anything*,* so why would I get anything? Even if they come up to the TCC*,* see it from outside but avoid stepping in.”* - TCC staff.

Most patients reaching the TCC are men, although nearly one-fourth of adult tobacco users are women in both districts. Uneducated and tribal patients ‘shy’ away from TCCs.

*“Tribal are uneducated; they get worried that something will happen*,* like an operation (for cancer) if they visit the TCC. They hide their faces when doctors ask. They feel ashamed and do not come here. They are (simple) they know that they are making mistakes when they consume tobacco. (and shy away from TCC)”*--District official.

All TB patients are ‘asked’ about tobacco use, but TB suspects or contacts do not necessarily undergo such screening. Few of the relatives of cancer patients are also ‘asked’ despite not being primary clients. The counsellors in the adolescent health programme (RKSK) receive referrals of tobacco-using adolescents from the OPD. However, not all adolescents are screened at the OPD, and adolescents usually do not turn up for follow-up.

### Fidelity

The common messages given by the service providers to the tobacco users are listed in Table 3. The messages are specific pieces of ‘advice’ to the type of patient. Most were individual one-to-one interactions. Group discussions were usually held at NCD clinics where patients visit regularly. The annual target of group discussions was achievable with quarterly group discussions at NCD clinics. Most of the messages were ‘Advice’ oriented. Very few healthcare providers provided illustrations of the next three As. Assessment of readiness was not prominent among the messages. Assisting messages were shared by NCD and TCC counsellors but not by other healthcare workers. The participants rarely spoke about ‘Arranging’ for follow-up. The key messages are shown in Box 1.

**Table 3:** ‘Advice’ Messages by type of client and provider interaction

Interaction	Messages
NCD patient – NCD counsellor	*If you continue consuming tobacco*,* then the medicine that you are taking will not be effective* *You will develop effects such as precancer*,* cancer*,* gangrene and amputation if diabetic* *You may not have felt the effects so far*,* but as age advances*,* you will start getting problems* *Show photos (and videos) of cancer patients and tell the real-life story of a patient from the same community*
Relatives of cancer patients – TCC staff	*How much did you have to rush and travel (for the cancer patient) and what is the risk in the future? What is your situation? You get an OPD paper and let us check your condition* *How many in your family consume tobacco? Pass the messages to them*
TB patient- TB worker	*If you will continue to smoke bidi*,* your lungs will weaken due to which*,* the side effects of anti-TB medicine are seen.* *If you continue to use tobacco*,* your medicines will not be effective* *Immunity has gone down due to tobacco use*,* and that is how you got TB*,* you should quit* *I show the patients fear - tobacco consumption leads to illnesses such as diabetes*,* hypertension and cancer; the largest risk is cancer*
Pregnant women – NCD counsellor, ANM	*It will adversely affect your health and also will have devastating effects on your baby’* *We tell them that we will visit your home*,* our sir may come* *If not for you*,* you need to quit for your baby* *If you use addictive substances*,* your child will also use these and the baby will be born malnourished (low-birth-weight)*
Husbands – NCD counsellor	*Keep a check on your wife’s tobacco consumption* *It (tobacco use) will harm the baby* *she (wife) will also get the habit if you consume tobacco*
Adolescents – RKSK counsellor	*Kharra consumption does not benefit (any) the body. There is no weight gain*,* there is no height gain.* *Kharra costs 30 rupees a day*,* i.e. 900 rupees a month. With 900 rupees you can buy a nice dress and shoes.* *Your friends are sharing Kharra with you. They may pay for your Kharra but if you get disease*,* will any friend share your disease burden?*
Tobacco user (General) – TCC staff	*Your 2-2.5lakh rupees have been spent on tobacco. With that*,* you could have made your house*,* and your children*’*s education could have been better* *Why do you need tobacco early in the morning whereas others do not need it? Because it has nicotine. It gets into saliva*,* reaches the brain through the blood and then affects…* *It has 4000 toxic chemicals (apart from nicotine). 70% of these chemicals are carcinogenic. Cancer is such that whichever part of the body gets cancer*,* that part has to be cut and thrown* *Hair loss*,* eye damage*,* cataracts*,* early ageing*,* teeth damage*,* foul-smelling breath*,* chances of heart attack*,* paralysis*,* kidney damage and gangrene. (Induce fear*,* it works)* *Lime burns skin. Compared to our outer skin*,* the mouth skin (oral mucosa) is so sensitive…It may lead to deformity and you will spend a lot of money. Maybe some part of your face needs to be cut and you may face difficulty in speaking and eating and even after spending money*,* you may not get good output (We frighten them… Some patients are a nuisance; they have to be made afraid of)*

Box 1

### Intensity

The intensity varies by patient type. In most cases, the intensity depended upon the planned visits associated with the patient’s underlying condition and is described in Table 4.

**Table 4:** Intensity of tobacco cessation received by the type of patient

Client	Intensity
Patient with NCD	Patients with NCD (Commonly hypertension and diabetes) are expected to visit monthly to collect their medicines. Patients who visit NCD clinics receive quit messages and counselling repeatedly. However, the services are also being decentralized beyond the NCD clinics (Secondary) to the HWCs (Primary) level. The ANM and ASHA reported that they only dispensed medicines and did not discuss tobacco cessation.
Relatives of cancer patients	No follow-up with relatives of cancer or other NCD patients
TB and HIV patients	Brief advice is certainly given at the beginning of treatment. Patients visit health facilities regularly for the duration of their treatment but the ‘brief advice’ is not necessarily repeated.
Pregnant women	As pregnant women access antenatal care periodically, brief advice is repeated throughout the antenatal period. The ANC services are often provided at the village level by ASHA and ANM who know the pregnant women.
Husbands of pregnant women	Lack of rigorous follow-up with husbands of pregnant women
Adolescents visiting clinics	Adolescents are usually referred from OPD to the adolescent clinics. Their primary conditions are often of short duration and the adolescents did not follow up at the clinics. The intensity was limited. None of the counsellors reported any case of adolescents reaching them for tobacco cessation.
Clients at TCC	Out of the 100–150 clients visiting *Nandurbar* TCC monthly, only 10% were follow-up cases. Comparatively, *Gadchiroli* TCC could follow-up 300 clients over the previous four months due to support from TMH. Anticipating the drop, the TCC staff suggests their clients to register with the quit line. No data on patients following up with quitline was available at the local TCCs
Pharmacotherapy	Not currently available in the public sector. Private practitioners and pharmacists did not maintain any tobacco cessation data. But a pharmacist reported ‘I have regular clients who come to buy NRT. They have been coming for months. It is also a kind of addiction, you see.’
Private sector	No data were available. Anecdotal evidence by practitioners who worked in both sectors said that tobacco cessation is neither the patient’s nor providers’ priority. The patient visits a private doctor for a health concern, and both aim to address that and tobacco may not get priority. The advice available in the private sector is largely NRT.

### Outcomes

The outcomes of the participants varied by age and gender. Aged patients were less likely to quit, and almost all reported fatalistic views. Counsellors reported that the common response of the elderly was ‘*My life is hardly left*,* it is almost over… I have lived my life. Now*,* if something happens*,* it is ok. If it does not happen*,* that is also ok.*’. The counsellors reported that young users younger than 30 years were more willing to quit. However, the adolescents were difficult clients. One adolescent reported, *‘With tobacco*,* one looks handsome. There are so many teachers in school who consume*,* many health workers consume*,* you are unnecessarily telling me that it causes cancer. (You are telling me a lie)’.* RKSK counsellors found it challenging to convince adolescents. *‘The age group is such that they are not in a frame of mind to listen. If we tell them*,* they act like they listen*,* they hear only for that time*,* but when they go home*,* there is no behaviour change. If we threaten*,* they will listen but for a limited time (temporary)*,* and then they will again start. They will come in contact with friends and restart.’-RKSK counsellor.* Follow-up was poor among adolescents, and relapse was common due to peer pressure, stress at the family level and break-up. Although access was poorer for women, their response was better. ‘*Responses are more or less the same in men and women. However*,* men*,* some say who is this guy to tell me? Women listen better.*’-TCC staff.

Quitting was common among the pregnant women. ‘*Since we have told them that it will affect their babies*,* if not for them but for the sake of their babies*,* they quit.’* was reported by providers. Providers said that women’s concerns become diluted after delivery. When they resume work on farms, they return to environments where many women use smokeless tobacco, and relapse is common. Some could abstain beyond the infancy stage of their babies.

TB workers reported that quit attempts were common in the first two weeks. However, those with high tobacco consumption cannot stop but reduce their consumption during the critical initial period. Drug-resistant TB patients are hospitalised for the first week, and the rules for inpatients prevent their use or substantially reduce their consumption. However, with treatment, symptoms decline, weight and general well-being improve, and fear disappears. Relapse was common, but data were not maintained. TB workers opined that only approximately one quarter or fewer of the tobacco users remained abstinent by the end of treatment, reducing the abstinent proportion to 10% after treatment completion.

Patients with cancer and other serious conditions were more likely to agree to quit advice. Those without major illnesses seldom return and do not respond favourably to telecalls. NCD and TCC counsellors reported that the fear of disease was a prominent facilitator.

*‘If the patient has oral fibrosis*,* leukoplakia*,* erythroplakia*,* or food looks very spicy*,* then the patient is afraid*,* and they move fast – many reach the last of 5As in the first session itself.’*-TCC staff.

Cancer patients and precancer patients had high rates of quitting attempts and the lowest risk of relapse. The NCD counsellors had impressions that approximately 25% to 30% of NCD patients quit; however, they were not maintaining any records. They expressed that group discussions and audio-visual aids in formal counselling sessions yield better results. works better than counselling alone. According to the TCC *Gadchiroli* records, the quit rate was approximately 10% (34 out of 326 quit for at least one month). The TCC staff mentioned that resolving to quit was very important, as tobacco had high dependence, and the social environment was not conducive to quitting. It is a condition of dependence. NRT was not offered, and there were no data on whether anyone had quit successfully or abstained for long durations from both tobacco and NRT.

## Discussion

Tobacco is a highly addictive substance, and provider-initiated cessation counselling is a critical initial step in generalising the will to quit among users. A recent commentary called for a Systems Approach to providing cessation services [14]. The commentary also highlights that there have been no district-level studies, as the scant cessation experience literature is from tobacco cessation clinics or premier academic institutes. The present study is one of the first at the district level to provide details of the district implementation model. Specialised tobacco cessation services were available in both districts with trained providers. The counsellors under the NCD programme have been trained and assigned NTCP responsibilities at the secondary healthcare level, thereby expanding the reach of cessation services beyond district TCCs.

Decentralisation of cessation services was envisaged, with the early promise shown in the TCCs. However, the integration of tobacco cessation services has been a concern since the beginning [20]. In the present study, primary healthcare facilities lacked dedicated human resources, which is understandable given the need to integrate services. Healthcare providers at the primary care level reported ‘asking’ patients about tobacco use, but mechanisms such as a ‘stamp’ on OPD paper are not operational at that level. Introducing these mechanisms may help in screening for tobacco use, major NCDs and other risk factors at the primary level. Tobacco use screening should be an integral part of overall NCD screening. Future studies should ‘quantify’ the screening for tobacco use at the primary care level, which will help improve this crucial component in cessation.

Community health workers are an important source at the village level. This study revealed that they were already screening pregnant women. A recent study reported the feasibility and acceptability of interventions using these methods [21]. However, the previous literature did not demonstrate high effectiveness in urban settings [22]. An effective intervention will expand reach to tobacco users spread across rural and tribal areas who would otherwise never reach the TCCs [23]. Although Community-Based Assessment Checklists (CBACs), which include tobacco use behaviours by individuals, are filled out by ASHAs (India’s community health workers), only those with a risk score above 4 are referred to health facilities. Daily tobacco users who do not have other risk factors are likely to score 4 and below and will not be prioritised for NCD screening. There is a need to quantify the proportion of tobacco users who are not getting prioritised for NCD screening and also understand whether the cessation behaviour is different among those who are referred to health facilities than among those who are not referred. There is indeed a need to shift focus from secondary prevention (diagnosis of NCDs) to primary prevention (tobacco cessation).

Tobacco use begins in the first or second decade for most users. The average age at initiation of daily tobacco use was 18.7 years among young adults aged 20–34 years [3]. The number of quit attempts is significantly lower among persons aged 65 years and above for smokeless tobacco [24]. The present study revealed that systematic screening at tertiary and secondary care facilities was limited to those older than 30 years, tuberculosis and HIV patients, and pregnant women. Younger patients were missed from the first ‘A’. Another concern with younger patients is that their health conditions are usually not chronic and that their follow-up is poor, even when some are referred to RKSK counsellors. A community-based approach is needed to connect adolescents with cessation services.

The second ‘A’ was dominated by messages that induce ‘fear’ to evoke the desire to quit among users. This was also true for pregnant women and tuberculosis patients at all levels. Healthcare providers reported relapse after delivery in pregnant women. We believe that this may be due to strong messages related to the adverse effects of tobacco on pregnancy outcomes, but a relative lack of messages that tobacco use is hazardous to overall health, with adverse effects on infants, young children, and future pregnancies in addition to the NCD burden in later years of life. TB workers also reported in this study that the initial willingness and abstinence from tobacco use waned by the end of treatment. There is a need to pilot-test specific messages for these groups and train primary care providers in evidence-based practice.

The ‘Assess’ and ‘Assist’ messages were uncommon at the primary care level. Capacity building by primary healthcare providers is also needed for the appropriate use of the next three ‘As’, and most stop after the second A (Advise). The TCC has a significant role in strengthening such capacities within the system. An inventory of standardised simple messages for assisting and assessing may be valuable. The time constraints of the general primary care provider must be considered, and job aids may be valuable. There is indeed a need for implementation research to improve the field-level effectiveness of the 5 ‘A’ approach.

Systematic data are being maintained only at TCCs, but cohort monitoring has not been institutionalised. Standardised cessation data will help understand the effectiveness of services at TCC and NCD clinics. It is possible that during routine service delivery, patients will provide desirable answers. Most studies have recorded self-reported data, which shows that a high proportion (up to 40%) of tobacco users who self-report cessation have biochemical samples showing cotinine. This affects the reliability of self-report as the sole method for assessing cessation [25]. NTCP should consider periodic cross-sectional studies with systematic sampling and biological assessments, such as urinary cotinine levels. Improved data management, supplemented by periodic sample surveys, will help us determine the actual effectiveness under program conditions. This study reported relapse after delivery among pregnant women and at treatment completion among TB patients. The measurement of success should take this into account.

It is possible to integrate cessation services in NCD care, which is expected to improve NCD outcomes as well, such as better control of hypertension and diabetes [15]. In the present study, the NCD counsellors reported during in-depth interviews that group sessions were helpful. However, they had not calculated the quit rates separately for those who attended group sessions and for those who did not. However, this cannot be backed by the data. Although the effectiveness of group counselling and self-help groups has potential, it has not been adequately tested in trials. There have been women’s movements against alcohol use [26], but such interventions against tobacco use have not been reported to our knowledge.

The follow-up rate reported at the TCCs was only 15% in a Bengaluru study [12]. There is a need to understand gaps in the cascade—between referrals to TCCs, the number of clients reached, the number of clients returning for follow-up, and the number of clients who can be followed up telephonically and remain abstinent. The effect of follow-up through telephonic or digital approaches on cessation counselling needs to be assessed through implementation research. Tobacco use by health workers can pose challenges to cessation services, and interventions should also address it [27].

The study had the following limitations. The study did not attempt to capture the patient’s perspective. However, it is an important area for investigation, and telephone or other means (e.g., following up with the departments that refer them) should be used. The two districts were purposively selected because of their high burden, but the type of tobacco used varied between them. Paan-masala with tobacco was the most consumed product in *Nandurbar*, with Paan-masala being sold in manufactured packets. *Gadchiroli* has a very high use of *Kharra*, which is freshly prepared by local vendors and sold. There is a likelihood of variation in product types from district to district. However, the TCC structure and other programmatic structures are uniform across the district, and we believe that the findings of this study also apply to other districts. The data presented in this paper is based on the narratives by the providers. The tobacco use data at the first visit is noted by TB, RMNCAH + N, HIV and NCD programmes, but no data on tobacco cessation attempts and sustenance is maintained by any of these programmes. Nevertheless, the insights shared by providers reveal the challenges in the cessation service process.

Recommendations: Decentralisation to the primary healthcare level is essential, and mechanisms that have been successful at the secondary care level, such as ‘stamp’ on the OPD paper, may be useful. CHOs need capacity-building in cessation counselling so that services reach as close to the community as possible. Strongly needed are supportive ‘advice’ messages and strengthening of ‘assess’ and ‘assist’ within the system. Systematic data on cessation need to be collected across all health programmes to capture the current status and constraints of tobacco cessation delivery.

## Conclusions

The tobacco cessation services have been decentralised from the district to lower levels, but their reach is limited. The capacity to provide quality counselling declines from district-level tobacco cessation centres to primary-level providers, and there is a need to increase primary-level providers’ capacity through brief key messages. The ‘fear-inducing’ tone is predominant in the ‘Advice’ messages, and the weaning of the efforts with the reduction of fear associated with other conditions, such as pregnancy and tuberculosis, is evident. The ‘Advice’ messages must be supplemented by ‘Assist’ and ‘Arrange’ approaches. Data on tobacco use should be collected periodically from clients utilising tobacco cessation services to help measure quit rates. Cessation outcomes should be systematically recorded in health service systems.

## Supplementary information

Below is the link to the electronic supplementary material.

Supplementary Material 1 Qualitative interview guides.

## Data Availability

The paper is based on primary data (in-depth interviews and focus group discussions) collected from the study participants and is saved with the first author in the form of audio files. The data summary is available upon request. The actual audio files cannot be shared due to privacy and confidentiality concerns.

## Abbreviations

AIDS: Acquired Immuno
ANM: Auxiliary Nurse Midwife
ASHA: Accredited Social Health Activist
CHC: Community Health Centre
CHO: Community Health Officer
DH: District Hospital
FCTC: Framework Convention for Tobacco Control
GATS: Global Adult Tobacco Survey
HIV: Human Immunodeficiency Virus
HWC: Health and Wellness Centre
LMICs: Low- and Middle-Income Countries
MC: Medical College
NCD: Non-communicable Disease
NFHS: National Family and Health Survey
NRT: Nicotine Replacement Therapy
NTCP: National Tobacco Control Programme
OPD: Out-Patient Department
PHC: Primary Health Centre
RCH: Reproductive and Child Health
RCT: Randomized Controlled Trial
RKSK: Rashtriya Kishor Swathya Karyakram
TB: Tuberculosis
TCC: Tobacco Cessation Clinic
WHO: World Health Organisation

## References

[1] He H, Pan Z, Wu J, Hu C, Bai L, Lyu J. Health Effects of Tobacco at the Global, Regional, and National Levels: Results From the 2019 Global Burden of Disease Study. NICOTINE Tob Res. 2022;24(6):864–70. 10.1093/ntr/ntab265. 34928373

[2] Reitsma MB, Kendrick PJ, Ababneh E, Abbafati C, Abbasi-Kangevari M, Abdoli A, et al. Spatial, temporal, and demographic patterns in prevalence of smoking tobacco use and attributable disease burden in 204 countries and territories, 1990–2019: a systematic analysis from the Global Burden of Disease Study 2019. Lancet. 2021;397(10292):2337–60. 10.1016/S0140-6736(21)01169-7. PMC8223261 34051883

[3] Tata Institute of Social Sciences. Mumbai, Ministry of Health and Family Welfare, Government of India. Global Adult Tobacco Survey GATS 2 India 2016-17. [Internet]. 2018 [cited 2023 Apr 8]. Report No. Available from: https://ntcp.mohfw.gov.in/assets/document/surveys-reports-publications/Global-Adult-Tobacco-Survey-Second-Round-India-2016-2017.pdf

[4] WHO. Development of an Implementation Roadmap 2023–2030 for The Global Action Plan for The Prevention and Control of NCDs 2013–2030. [Internet]. WHO. 2021. Available from: https://cdn.who.int/media/docs/default-source/documents/health-topics/non-communicable-diseases/eb150---who-discussion-paper-on-ncd-roadmap-development-(20-aug-2021)---for-web.pdf?sfvrsn=58b8c366_17&download=true

[5] United States Public Health Service Office of the Surgeon General, Health NC for CDP and HP (US) O on S and. The Health Benefits of Smoking Cessation. In: Smoking Cessation: A Report of the Surgeon General [Internet] [Internet]. US Department of Health and Human Services. 2020 [cited 2024 Sep 3]. Available from: https://www.ncbi.nlm.nih.gov/books/NBK555590/

[6] WHO. WHO report on the global tobacco epidemic, 2023 Protect people from tobacco smoke [Annual]. Geneva. 2023. Report No.

[7] Kumar N, Janmohamed K, Jiang J, Ainooson J, Billings A, Chen GQ, et al. Tobacco cessation in low- to middle-income countries: A scoping review of randomized controlled trials. Addict Behav. 2021;112:106612. 10.1016/j.addbeh.2020.106612. 33002679

[8] Gupta R, Verma V, Mathur P. Quitline Activity in Rajasthan, India. Asian Pac J Cancer Prev APJCP. 2016;17(S2):19–24. 10.7314/apjcp.2016.17. .s2.19 PubMed PMID: 27108749. 27108749

[9] Kumar M, Kumar N, Gupta P, Kumar S, Mishra J, Kumar R, et al. National Tobacco Quitline – A Comparative Study of Prevalence of Smoking and Smokeless Tobacco Use in India: A Brief One-year Report. Indian J Chest Dis Allied Sci. 2018;60(4):221–5. 10.5005/ijcdas-60-4-221.

[10] Pahwa V, Pimple SA, Bhattacharjee A, Kuberkar D, Mishra GA, Chaturvedi P. Behavioural interventions for tobacco cessation in India: A systematic review and meta-analysis. J Fam Med Prim Care. 2023;12(11):2542–51. 10.4103/jfmpc.jfmpc_1017_23. PMC10771179 38186806

[11] Kaur J, Jain D. Tobacco Control Policies in India: Implementation and Challenges. Indian J Public Health. 2011;55(3):220. 10.4103/0019-557X.89941. 22089690

[12] Mony PK, Rose DP, Sreedaran P, D’Souza G, Srinivasan K. Tobacco cessation outcomes in a cohort of patients attending a chest medicine outpatient clinic in Bangalore city, southern India. Indian J Med Res. 2014;139(4):523–30. PubMed PMID: 24927338; PubMed Central PMCID: PMC4078490. PMC4078490 24927338

[13] Gupta R, Bhatt G, Narake S, Udawat H, Goel S. Outcomes of Integrating Quitline Methodology in Tobacco Cessation Delivered through a Model Tobacco Treatment Clinic of a Private Sector Hospital at Rajasthan, India. Indian J Public Health. 2021;65(3). 10.4103/ijph.IJPH_151_21 34558492

[14] Gupta R, Aghi MB, Gupta A, Bhatt G, Goel S. Strategic Initiatives to Improve Tobacco Cessation Delivery in India. Indian J Community Med. 2022;47(3):328–31. 10.4103/ijcm.ijcm_1399_21. PMC9693939 36438508

[15] Bhatt G, Goel S, Grover S, Medhi B, Jaswal N, Gill SS, et al. Feasibility of tobacco cessation intervention at non-communicable diseases clinics: A qualitative study from a North Indian State. PLoS ONE. 2023;18(5):e0284920. 10.1371/journal.pone.0284920. PubMed PMID: 37141319; PubMed Central PMCID: PMC10159160. PMC10159160 37141319

[16] Bhatt G, Goel S, Yadav SK, Patial A, Medhi B, Grover S, et al. A randomised controlled trial to evaluate the effectiveness of a culture and disease-specific, patient-centric multi-component tobacco cessation intervention package for the patients attending non-communicable disease clinics in Punjab, India. Psychol Health. 2024;1–18. 10.1080/08870446.2024.2333896. PubMed PMID: 38619313. 38619313

[17] Shankar A, Parascandola M, Sakthivel P, Kaur J, Saini D, Jayaraj NP. Advancing Tobacco Cessation in LMICs. Curr Oncol. 2022;29(12):12. 10.3390/curroncol29120713. PMC9777415 36547127

[18] Gupta R, Pednekar MS, Kumar R, Goel S. Tobacco cessation in India–Current status, challenges, barriers and solutions. Indian J Tuberc. 2021;68:S80–5. 10.1016/j.ijtb.2021.08.027. 34538397

[19] Linnan L, Steckler A. Process Evaluation for Public Health Interventions and Research: An overview. In: Process Evaluation for Public Health Interventions and Research. 2002.

[20] Varghese C, Kaur J, Desai N, Murthy P, Malhotra S, Subbakrishna D, et al. Initiating tobacco cessation services in India: challenges and opportunities. WHO South-East Asia J Public Health. 2012;1(2):159. 10.4103/2224-3151.206929. 28612792

[21] Gupte HA, D’Costa M, Gupta S, Sonawane V. Integration of a Tobacco Cessation Program into a Rural Community-Based Maternal and Child Health Program in India: A Stakeholders’ Perspective on Task Shifting. Nicotine Tob Res. 2024;26(9):1209–17. 10.1093/ntr/ntae058. 38477033

[22] Sarkar BK, West R, Arora M, Ahluwalia JS, Reddy KS, Shahab L. Effectiveness of a brief community outreach tobacco cessation intervention in India: a cluster-randomised controlled trial (the BABEX Trial). Thorax. 2017;72(2):167–73. 10.1136/thoraxjnl-2016-208732. PMC5284331 27708113

[23] Jiang N, Siman N, Cleland CM, Van Devanter N, Trang N, Nam N, et al. Effectiveness of Village Health Worker-Delivered Smoking Cessation Counseling in Vietnam. NICOTINE Tob Res. 2019;21(11):1524–30. 10.1093/ntr/nty216. PMC6941703 30335180

[24] Gawde NC, Syed ZQ. Determinants of quit attempts and short-term abstinence among smokeless tobacco users in India: Global Adult Tobacco Survey, 2016-17. 10.31557/APJCP.2024.25.3.875 PMC11152389 38546070

[25] Scheuermann TS, Richter KP, Rigotti NA, Cummins SE, Harrington KF, Sherman SE, et al. Accuracy of self-reported smoking abstinence in clinical trials of hospital-initiated smoking interventions. Addiction. 2017;112(12):2227–36. 10.1111/add.13913. PubMed PMID: 28834608; PubMed Central PMCID: PMC5673569. PMC5673569 28834608

[26] Pande R. From anti-arrack to total prohibition: the women’s movement in Andhra Pradesh, India. Gend Technol Dev. 2000;4(1):131–44. doi:10.1177/097185240000400109 PubMed PMID: 12179945. 10.1177/097185240000400109 12179945

[27] Abdullah AS, Stillman FA, Yang L, Luo H, Zhang Z, Samet JM. Tobacco Use and Smoking Cessation Practices among Physicians in Developing Countries: A Literature Review (1987–2010). Int J Environ Res Public Health. 2014;11(1):429–55. 10.3390/ijerph110100429. PMC3924453 24380976

