# Supplementary material for: Exploring the implementation of tobacco cessation services in government healthcare facilities, Maharashtra: a qualitative approach
Source: BMC Health Serv Res. 2026 Apr 17;26:1541. doi: 10.1186/s12913-026-14499-x (PMC13741624; doi:10.1186/s12913-026-14499-x)
Supplement: Supplementary file 1 [file 12913_2026_14499_MOESM1_ESM.docx]

**In-depth Interview guide for programme managers/ service providers**

1. Tell me about your designation, roles and responsibilities especially with tobacco control
2. Tell us about the interventions under the National Tobacco Control Programme within the state/ district over past decade. (Probes: When was NTCP started, how many districts were involved, types of facilities, types of interventions)
3. What human resources have been involved for delivery of NTCP and what have been challenges? (probes: vertical or integrated models of care, challenges for availability and skills of the human resources)
4. Tell us about financial resources for the interventions. (Probes: Sources of finance, sufficiency and continuum in finance, manner in which it has affected delivery of services)
5. Tell us about other resources for interventions (Probes: types of resources such as materials, drugs, etc.; their supply chain management issues)
6. Tell us specifically about interventions of the government to help users in quitting (probe for advertisement in media, warning on labels, pharmacotherapy, counselling, quit-line, etc.)
7. What have been reach of these interventions? (Probes: number of people reached, districts covered by interventions, data on people who have quit)
8. How is COTPA implemented in the state/ district? (probe: mechanisms to implement COTPA and who are the law enforcers, data on actions taken – fines for individual users, restaurant/ public place owners/ manufacturers/ advertisers/ sellers)
9. What are the price related regulations of tobacco in your state/district? Which products are under (and which are not) regulations? (Probes: price regulations by the state, ban on certain products, ban on sell of loose cigarettes, etc.)
10. According to you which interventions for tobacco control have worked the best? (Probes: which interventions have worked and why? Which interventions did not work and why did they not work? Are individual interventions more successful or mass media interventions?)
11. What are your suggestions to improve tobacco control activities especially cessation of tobacco use?

**In-depth Interview guide for other stakeholders**

1. Tell me about your designation, roles and responsibilities within your organisation especially with tobacco control (Probe: type of organisation and responsibility – advocacy, implementation of COTPA, research, etc.)
2. Tell us about the interventions under the National Tobacco Control Programme within the state/ district over past decade. (Probes: When was NTCP started, how many districts were involved, types of facilities, types of interventions)
3. What has been role of your organisation in tobacco control in the state? (probes: role of organisation, work at policy/ field level, work along with government or for government or independent of government)
4. Tell us specifically about interventions of the government to help users in quitting (probe for advertisement in media, warning on labels, pharmacotherapy, counselling, quit-line, etc.)
5. What have been reach of these interventions? (Probes: number of people reached, districts covered by interventions, data on people who have quit)
6. How is COTPA implemented in the state/ district? (probe: mechanisms to implement COTPA and who are the law enforcers, data on actions taken – fines for individual users, restaurant/ public place owners/ manufacturers/ advertisers/ sellers)
7. What are the price related regulations of tobacco in your state/district? Which products are under (and which are not) regulations? (Probes: price regulations by the state, ban on certain products, ban on sell of loose cigarettes, etc.)
8. According to you which interventions for tobacco control have worked the best? (Probes: which interventions have worked and why? Which interventions did not work and why did they not work? Are individual interventions more successful or mass media interventions?)
9. What are your suggestions to improve tobacco control activities especially cessation of tobacco use?
